# Does an expert-based evaluation allow us to go beyond the Impact Factor? Experiences from building a ranking of national journals in Poland

**DOI:** 10.1007/s11192-017-2261-x

**Published:** 2017-02-01

**Authors:** Emanuel Kulczycki, Ewa A. Rozkosz

**Affiliations:** 1 Adam Mickiewicz University in Poznań, Poznań, Poland; 2 University of Lower Silesia, Wrocław, Poland

**Keywords:** Journal ranking, Multidimensional evaluation, Structural equation modelling, Expert-based evaluation, Citations, Poland, O38, 62-07

## Abstract

This article discusses the Polish Journal Ranking, which is used in the research evaluation system in Poland. In 2015, the ranking, which represents all disciplines, allocated 17,437 journals into three lists: A, B, and C. The B list constitutes a ranking of Polish journals that are indexed neither in the Web of Science nor the European Reference Index for the Humanities. This ranking was built by evaluating journals in three dimensions: formal, bibliometric, and expert-based. We have analysed data on 2035 Polish journals from the B list. Our study aims to determine how an expert-based evaluation influenced the results of final evaluation. In our study, we used structural equation modelling, which is regression based, and we designed three pairs of theoretical models for three fields of science: (1) humanities, (2) social sciences, and (3) engineering, natural sciences, and medical sciences. Each pair consisted of the full model and the reduced model (i.e., the model without the expert-based evaluation). Our analysis revealed that the multidimensional evaluation of local journals should not rely only on the bibliometric indicators, which are based on the Web of Science or Scopus. Moreover, we have shown that the expert-based evaluation plays a major role in all fields of science. We conclude with recommendations that the formal evaluation should be reduced to verifiable parameters and that the expert-based evaluation should be based on common guidelines for the experts.

## Introduction

Several countries have established performance-based research funding systems (PRFSs). A PRFS can be based on either a peer review or on an indicator-based model (Hicks 2012). The *Research Excellence Framework* in England is the most well-known example of a peer review model (Wilsdon et al. 2015). Indicator-based models have been implemented in Flanders (Belgium), Italy, Nordic countries, and Poland, among others. These indicator-based models provide classifications for publication channels in terms of the publications’ quality. In the Nordic system and in Flanders, full coverage national databases for recording and validating academic publications have been developed (Verleysen et al. 2014). In Italy, publication quality is assessed through a combination of citations and journal metrics (Abramo and D’Angelo 2016). In Finland, the characteristics of all relevant publication channels are provided by the *JuFo* database (Saarela et al. 2016). The Polish database, *Polish Scholarly Bibliography,* records all publications by Polish scholars since 2009.

Much has been written about the journal rankings and journal classifications (Hodge and Lacasse 2011; Serenko and Dohan 2011). On the one hand, journal rankings are useful tools for researchers, stakeholders, and policy makers. Scholars publishing in high-ranked journals receive higher salaries (Gomez-Mejia and Balkin 1992). Various higher-education institutions have implemented policies for rewarding their faculty for publishing in top-tier journals (Manning and Barrette 2005). Publishing in the high-ranked journals is perceived as one of the most important aims of science. On the other hand, there are many controversies surrounding the rating of journals. Most of them focus, among others things, on the ranking impact on academic life (Brembs et al. 2013; Reuter 2011; Wheeler 2011), the marginalisation of non-English countries (Wolters 2013), the relation of the citation metrics to the expert judgment (Haddawy et al. 2016; Sangster 2015; Serenko and Dohan 2011), and building a ranking for the social sciences and humanities (SSH) journals (Ferrara and Bonaccorsi 2016).

The two most often used methods of constructing journal rankings have relied on expert-based and citation-based evaluation. Serenko and Dohan (2011) compared various rankings based on both the expert surveys and the citation-based measures. They conclude that these two methods cannot be used as theirs substitutes. An expert-based evaluation and an evaluation based on the journal-citation measures should instead be used as complementary approaches. Saarela et al. show that based on the Finnish *JuFo* system, most expert-based rankings can be predicted and explained using automatically constructed reference models. Thomas and Watkins (1998) claim that the expert surveys and the citation-based measures strongly correlate, whereas Maier (2006) points out no significant positive correlation between the Impact Factors and the peer judgments. Schloegl and Stock (2004) state that the ranking lists, based on these two approaches, are very inconsistent. Nonetheless, in light of the present paper aim, it is worth highlighting that most rankings using these two approaches relied on the journal citation measures based on the Web of Science (WoS), Scopus, and Google Scholar (Ahlgren and Waltman 2014; Bontis and Serenko 2009; Haddow and Genoni 2010; Pajić 2015; Vanclay 2008). However, these rankings are often limited to some narrow fields or disciplines, such as forestry journals (Vanclay 2008) or Taiwanese journals in the SSH (Kao et al. 2008).

In addition to national databases, in which publications are recorded and validated, various journal rankings have been carried out at the national level. In France, Agence d’Evaluation de la Recherche et de l’Enseignement Superieur published a list of 6305 journals organised into three categories. In Australia within the Excellence in Research for Australia, an expert-based classification of more than 20,000 journals was published in 2010. Currently, the Australian list (i.e., the ERA 2015 Submission Journal List) consists of more than 16,000 journals. In Serbia, national journals are ranked and categorized annually in the Journal Bibliometric Report (Šipka 2013). Other national solutions have also been implemented in Norway, Taiwan, Brazil, Colombia, and the Netherlands. It is noteworthy to mention the international experience on the classification journals (i.e., the European Reference Index for the Humanities [ERIH]), which was built by the European Science Foundation in 2001 and evolved over various cycles (Wolters 2013). Finally, the ERIH was transferred to the Norwegian Centre for Research Data and has become a part of the ERIH PLUS.

In Poland since 2009, the research output has been assessed using a new system—the Comprehensive Evaluation of Scientific Units—developed by the Ministry of Science and Higher Education in Poland (Koczkodaj et al. 2014; Kulczycki 2017). An important component of this system is the Polish Journal Ranking (PJR) that allocates journals into three lists—A, B, and C—which translates the ‘quality’ of articles published in these journals into ‘the points’. National and non-national journals across all disciplines are represented in the ranking, similarly to, for example, the Australian, French, and Spanish systems (Ferrara and Bonaccorsi 2016; Haddow and Genoni 2010). A PJR, annually prepared by the Specialist Team for the Evaluation of Scientific Journals (STESJ), has a major impact on how the higher education institutions and researchers are financially and reputationally rewarded.

PJRs are built for and used in scientific unit funding decisions. Publication in a journal is the most important parameter of evaluation for which a scientific unit could possibly obtain the highest number of points. In the last cycle of evaluation in 2013, the percentage of funding depending on the results of evaluation ranged from 4 to 22% of the statutory funding for higher education institutions. PJRs are also used in promotion procedures. A Polish law required that only publications in journals indexed on the PJRs might be considered in decisions on a habilitation (the highest scientific degree). According to Moya et al. (2015), such a situation resembles the Spanish and Romanian solutions. In Poland, a candidate for a degree can list other publications, however, those that are not included in the government-controlled list ‘do not count’. Moreover, some universities require that a candidate collects a certain number of points (e.g. 250 points) and their h-index should be, for example, at least 3, if they want to obtain a habilitation. This is not a government criterion but a university recommendation or a prerequisite for those who want to start the procedure. However, these recommendations are explicitly expressed in terms of the points that can be obtained by publishing in journals indexed in the PJRs (Kulczycki 2017).

Nearly 3000 scientific journals have been published in Poland of which 2477 are indexed in the 2015 PJR. The remainder of the journals that do not fulfil the PJR criteria—for instance, they are published non-periodically—are indexed in the ARIANTA, which is the largest continuously updated Polish journal reference list. Currently in the WoS, however, there are only 231 Polish journals indexed. These include the Journal Citation Reports (JCR): 137, Arts and Humanities Citation Index: 6, and Emerging Sources Citation Index: 88. In SCOPUS, there are 350 journals indexed, and there are 139 Polish journals included in the ERIH. Because the PJRs are used for evaluating all Polish higher-education institutions, the local publications that are not indexed in the international databases must be taken into account. Thus, a national journal ranking in Poland, which should represent all disciplines, could not be based only on the Impact Factor or other bibliometrics indicators, which are not suitable for assessing, for example, publications in the humanities. The degree of coverage in the SSH in the Web of Science, which is the only government-acknowledged database in Poland, is low (Sivertsen and Larsen 2012), particularly for non-English publications.

The regulations that determine the PJRs have initiated many discussions and controversies. In Poland, there are much fewer publications in English than in other non-English countries such as Norway. According to Sivertsen (2016), in Norway, humanists published 61.1% of their publications in international languages and in the social sciences, the value of this indicator was even higher—71.7%. Sivertsen concludes that researchers are normally bilingual in their publication practices in the SSH. We cannot directly compare Norwegian and Polish practices because of the different levels of data collection. Sivertsen presents data at the level of individual researchers, whereas in Poland, data are collected at the level of all higher education institutions and at the level of Polish journals. In the 2009–2012 period, Polish scholars in all fields of sciences published and submitted 19,764 monographs to the Comprehensive Evaluation of Scientific Units, of which 86.7% were published in Polish; in the SSH, this value was slightly higher—87.8%. There were 144,873 book chapters published, of which 77.4% were published in Polish; in the SSH—80.2%. According to the Polish journals not indexed in the JCR or in the ERIH (*N* = 2035), the mean percentage of the articles published in international languages is 26% for the social sciences, 26% for the humanities, and 46% for the so-called hard sciences. These numbers show that Polish scholars publish mostly in Polish.

Until 2014, the PJRs were based on the two dimensions of evaluation: the formal and the bibliometric. In 2015, the STESJ, taking the criticism into account, decided to add the other dimension (i.e., the expert-based dimension, but only for the evaluation of national journals that are not indexed in the JCR or the ERIH).

Vieira and Gomes (2015) show that a bibliometric evaluation relates to an expert-based evaluation in three different ways: (1) bibliometrics are used for analysing the expert-based evaluation, (2) the expert-based evaluation uses bibliometrics as an auxiliary tool, and (3) the expert-based evaluation is a way of correcting the results of the bibliometric assessment. These methods have been implemented in various national models. For example, in Norway (as well as Denmark and Finland, which follow the Norwegian model), citation-based indicators are first collected and then presented to experts who can make their judgment based on them as well as other available journal information. In contrast, in the Polish system, the experts are provided neither with citation-based indicators nor the formal evaluation of the journal. According to this classification, the third relation characterises the case of the 2015 PJR.

The aim of the present paper is to determine how the expert-based evaluation influenced the results of final evaluation (2015 PJR). In our analysis, we tested three models designed separately for three fields of science: the humanities (H), the social sciences (SS), and the engineering, natural and medical sciences (ENM).

Like every scientometric tool, a PJR has some drawbacks and advantages (the most important are characterised in the present paper). It is noteworthy that building such a national ranking in a non-English country is the most important value for scholars and stakeholders. Thus, a PJR should and could be developed. We have conducted our research to show how to improve the PJRs and reduce the drawbacks resulting from overregulation and using incorrect bibliometric indicators.

The present study achieves this aim by examining the results of journal evaluation within the formal and bibliometric dimensions and comparing these findings with the results that also include the expert-based dimension of evaluation. Our study contributes to the discourse on the value and the utility of bibliometric indicators and has particular relevance to journal evaluation from non-English countries. The present paper also addresses several important and related issues that arise when considering the implication of building journal ranking for all fields of science. More precisely, this study aims at answering the following research questions regarding building a ranking of national journals:

1. What theoretical models demonstrate a good fit to the data of the multidimensional evaluation of national journals in Poland?
2. How has the expert-based evaluation influenced the results of the multidimensional evaluation of national journals in Poland, that is, the points? In other words: What effect would removing the expert-based evaluation have on the current version of journal evaluation system?

We deem these questions important because:

- Only a small part of non-English journals is indexed in the most often used databases (i.e., the WoS and SCOPUS) in research-evaluation systems.
- There is a need to rank journals without using the Impact Factor as the main indicator.
- The ongoing discussion on using the bibliometric indicators as predictors of the expert judgments needs arguments that rely on the experiences from various scholar cultures.

The paper is structured as follows: in “The 2015 PJR” section, we present the framework of the 2015 PJR and explain the evaluation process. In “Material and Methods” section, the Materials and methods are described, and the next section presents the results, focusing on the theoretical models designed for the three fields of science (H, SS, and ENM). In the final section, we discuss the main findings and conclusion.

## The 2015 PJR

The 2015 PJR allocates 17,437 journals into three lists—A, B, and C. The set of A, B, C lists makes up a national (Polish) journal ranking. An isolated list (i.e., the *B list*) constitutes a ranking of Polish journals, the structure and regulations of which will be examined in the present paper. The *B list* plays a major role in the science policy in Poland because Polish scientists have published more frequently in journals indexed in the *B list* than in journals indexed in the *A* or *C lists*.

The STESJ built the 2015 PJR according to *Komunikat Ministra Nauki i Szkolnictwa Wyższego z dnia 2 czerwca 2015 r. w sprawie kryteriów i trybu oceny czasopism naukowych* (the Regulation of the Ministry of Science and Higher Education of 2 June, 2015 on criteria and procedure of the evaluation of scientific journals). There were two technical operators that supported the STESJ work: the National Information Processing Institute and the Index Copernicus International.

The number of points assigned to a journal included in the 2015 PJR depended on the following regulations:

1. *The A list* (11,114 journals): the rank-normalised the five-year impact factor that was translated into the number of points (15, 20, 25, 30, 35, 40, 45, and 50). This number depended on the five-year impact factor values in the JCR subject category. The normalisation was provided separately for each subject category in the JCR (e.g. ‘Communication’ or ‘Acoustics’). Therefore, two journals with different numbers for their Impact Factors could be assigned the same number of points.
2. *The B list* (2212 journals): only Polish journals that were not indexed in the JCR and fulfil the entrance criteria (see Table 1) were included. There were three dimensions of evaluation: formal, bibliometric, and expert-based. The number of points (from 1 to 15) depended on how many parameters were fulfilled in each dimension (see Table 2).

**Table 1:** The entrance criteria for the *B list* of the 2015 PJR

Criterion	Requirement
List of reviewers	Publishing a list of reviewers at least once a year
Peer review process	Using a review policy that is published on a journal website: the double-blind review is recommended
Up-to-date website	Updating a journal website and publishing (at least) instructions for authors, editorial policies, and contents
External reviewers	Reviewing at least 50% of articles by the external reviewers, which are defined as the reviewers outside the editorial board and the institution in which an editor-in-chief is affiliated
Abstracts and titles	Publishing a title and an abstract in English for all articles
Continuity of publishing	Publishing all issues without postponements greater than 6 months

**Table 2:**
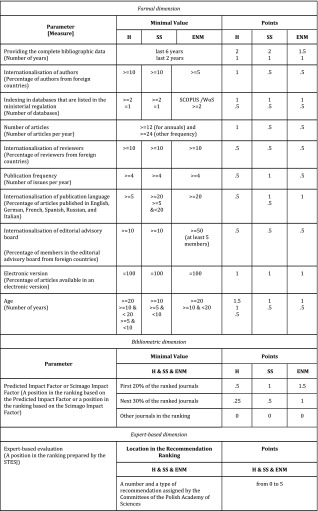
Parameters and minimal values in the evaluation within the *B list* of the 2015 PJR according to the fields of science: humanities (H), social sciences (SS), and the engineering, natural, and medical sciences (ENM)
3. *The C list* (4111 journals): only journals indexed in the ERIH were included. The number of points (10, 15, 20, and 25) depended on a bibliometric indicator based on the Scimago Journal and Country Rank.

All Polish journals had to submit the *Ankieta czasopisma naukowego* (The Questionnaire of Scientific Journal) through the *Polska Bibliografia Naukowa* (the Polish Scholarly Bibliography) to be indexed on the *B list* of the 2015 PJR. A Polish journal is defined as a journal for which the editorial office or the publishing house is located in Poland. Moreover, a journal had to be published at least for two years: data from the last two years were submitted through the questionnaire. Both the questionnaire and the final evaluation of each journal in all dimensions have been published and available on the *Polska Bibliografia Naukowa.*

The *B list* was built in two steps in which: (1) a fulfilment of the entrance criteria was verified, and (2) fulfilment of the required minimal values of parameters was verified.

In Step 1, each journal had to fulfil at least five of six entrance criteria to go to Step 2. As Table 1 shows, the entrance criteria were formal and related to the editorial policies and standards.

In Step 2, each journal was assessed within three dimensions of the evaluation: formal, bibliometric, and expert-based. As Table 2 shows, various parameters were defined and required minimal values for the fulfilment of these parameters. There is a single exception in the expert-based dimension in which the value of the only parameter, that is, the expert-based evaluation, is equal to the points obtained for it. This means that fulfilment of this parameter allows the journal to get 1–5 points.

During a submission of the above-mentioned questionnaire, a journal editor must choose one of the three fields of science in which a journal should be evaluated (i.e., the H, the SS, or the ENM). Moreover, a journal editor must indicate at least one and at most three disciplines that characterise a scientific profile of the published articles. For all fields of science, the same sets of parameters were designed. According to the field, however, different required minimal values were assigned for the fulfilling parameter, and a number of points were assigned for it. Points obtained by fulfilling the parameters are totalled. This sum of the points provides the result of the multidimensional evaluation of a given journal.

To illustrate how required minimal values could differ as a result of fulfilling the parameters, let us examine two cases. The first case concerns points for fulfilling the same parameter in different fields of the sciences. For instance, a journal from the H could obtain 1 point if at least 5% of articles in the evaluated period were published by the authors from foreign countries. Conversely, a journal from the SS or the ENM could obtain only a half point and only when at least 10% of the articles were published by the authors from foreign countries. The other case concerns journals from the same field of the sciences that have similar parametric values but have obtained different numbers of points because of the required minimal values. For instance, 9% of authors from the H in the first journal are from foreign countries and 10% of authors from the H in the second journal are from foreign countries. The second journal obtains 1 point for the ‘Internationalisation of authors’ parameter and the first journal receives no points for this parameter because of the required minimal values (10% in the H).

*Formal dimension* was based on the data submitted through the questionnaire. Moreover, journal editors could provide the bibliographic data to the POL-index. This database has been designed for collecting the references from all articles published in the Polish journals. The Ministry of Science and Higher Education has announced that, on the basis of the POL-index, the *Polish Impact Factor* will be calculated (orig. Polski Współczynnik Wpływu) in the next cycles of journal evaluation, probably in 2017. The STESJ verified the data quality submitted by various journals, which were pointed out by the technical operators and the STESJ members. The editors were not obligated to send electronic or paper versions of the evaluated issues during the questionnaire submission. Therefore, preparing the final formal ranking was founded on trust in the provided information by the journal representatives.

*Bibliometric dimension* was based on two bibliometric indicators: *Predicted Impact Factor* (PIF) and *Scimago Impact Factor* (SIF). On the *B list,* only journals without an Impact Factor could be indexed. In addition, SCOPUS is not an acknowledged database in the Polish science policy. However, to implement some bibliometric assessment within a ranking of national journals, the STESJ has proposed the two indicators in question.

PIF for a journal was calculated on the basis of the WoS (searched through the ‘Cited Reference Search’ form), and it was calculated for all journals that were submitted to the *B list* (*N* = 2212), regardless of whether they were indexed in the WoS. The PIF indicator was defined as follows:

$${\text{Predicted Impact Factor}}_{ 2 0 1 5} = A /B$$

where *A* the number of times that all items published in that journal (regardless of publishing year) were cited by indexed publications during 2012–2014. *B* the total number of articles published by that journal in 2012–2014 and indicated in the questionnaire.

SIF for a journal was calculated on the basis of the Scimago Journal and Country Rank, and it was calculated only for the Polish journals that were indexed in SCOPUS (*N* = 157). SIF for a journal was defined as:

$${\text{Scimago Impact Factor}}_{ 2 0 1 5} = C /B$$

where *C* the value of total cites (3 years), *B* the total number of articles published by that journal in 2012–2014 and indicated in the questionnaire.

During the evaluation, the Library of the University of Silesia in Katowice collected the data, and then the technical operators calculated PIF and SIF for the submitted journals. If a journal had calculated both SIF and PIF, a higher value of indicators was taken into account for a given journal in the final bibliometric ranking prepared in the bibliometric dimension.

We have examined the percentage of journals from the three fields of science that have a value of the SIF and PIF greater than or equal to zero. In the evaluation procedure, it was assumed that if a journal is not indexed in SCOPUS, then this journal has a SIF equal to zero. It allowed us to decide how many Polish journals can be evaluated through the bibliometrics indicators from the WoS or SCOPUS. Table 3 presents the results of our examination.

**Table 3:** PIF and SIF values according to the field of science: humanities (*N* = 734), social sciences (*N* = 680), and the engineering, natural and medical sciences (*N* = 798)

Value of a bibliometric indicator	Humanities		Social sciences		Engineering, natural and medical sciences	
	*N*	%	*N*	%	*N*	%
PIF > 0	349	48	329	48	716	90
PIF = 0	385	52	351	52	82	10
SIF > 0	8	1	14	2	134	17
SIF = 0	726	99	666	98	664	83
PIF > 0 or SIF > 0	349	48	329	48	717	90

We have found that using SIF and PIF will allow us to evaluate 90% of journals from the ENM, but in the two other fields of science, the percentage is significantly lower (i.e., 48% for the SS and 48%) for the ENM. Thus, the multidimensional evaluation should not be reduced only to the bibliometric evaluation if it has to serve for assessing journals from all fields of science: too many journals (52%) from the SS and the H could be omitted in the evaluation because of a lack of citations in the WoS or SCOPUS. Our findings confirm that journals in the SSH are underrepresented in the major citation databases (Sivertsen 2016).

*Expert*-*based dimension* involved the Committees of the Polish Academy of Sciences. Committees represent the Polish scientific community according to a discipline (e.g., the Committee on Psychological Sciences and the Committee on Geodesy). A technical operator of evaluation sent committees a list of journals that best fit the profile of a given committee. The process of assigning journals to committees was conducted on the basis of indicated discipline(s) in the submitted questionnaire by a journal and on the basis of the committee’s scientific profile. However, during a process of the evaluation, committees may have to add other journals if they decide that they are qualified to assess the journals in question. In this way, two committees received more than 500 journals for evaluation, 41 committees received more than 100 journals, 46 committees received from 10 to 100 journals, and only 5 committees received less than 10 journals. Finally, 94 committees prepared the expert recommendations.

Each committee could decide how many experts to invite to the evaluation process. Moreover, experts could be external (i.e., not members of a committee). The regulations regarding how to proceed this evaluation were designed by each committee. The STESJ specified only one guideline: each committee was asked to recommend, on the basis of the set of assigned journals, at most 10% of the journals that the experts (appointed by a given committee) acknowledged as ‘outstanding’ and to recommend at most 10% as ‘significant’. Finally, 1367 journals were acknowledged as ‘outstanding’ or ‘significant’ by at least one committee. On the basis of the expert-based evaluation prepared by all committees, the STESJ assigned the points to journals. Taken into consideration were the types and the number of recommendations for a given journal (an ‘outstanding’ recommendation was more important than a ‘significant’ one). Some factors that could disturb the evaluation and the results comparison between the committees include access to the previous evaluation results (if some journal was in the previous PJRs, it might mean that is ‘good enough’ to be recognised as a good one), an alphabetic order of the journals indicated for the evaluation, a different number of experts in the various committees (from one to over a dozen), and the committees’ diligence (some committees did not conduct their duties and recommended no journals).

## Materials and methods

### Material

In the present study, we used the data from the 2015 PJR, which was delivered by the National Information Processing Institute. The dataset contains: (1) the data submitted by the journals through the Questionnaire of Scientific Journals, (2) the fields of science in which journals were evaluated, (3) a number of points obtained for the fulfilment of each parameter by each journal, (4) a number of points obtained for the expert-based evaluation, and (5) the final point rank (i.e., a number of points assigned to each journal as the result of the multidimensional evaluation).

We have divided the data into three sets according to the field of science: the H, the SS, and the ENM. This step of the analysis emerges from differences between the minimal values and a number of points obtained for the fulfilment of the parameters in particular fields. The analysis within the separate fields allows us to highlight the differences in parameter fulfilment and to indicate fields that could not be evaluated through the bibliometric indicators.

The dataset consists of all evaluated journals (*N* = 2212), which are indexed in the *B list*, including 798 in the H, 680 in the SS, and 734 in the ENM. The unit of analysis is a journal. Data collection was performed in December 2015. We reduced the dataset by excluding 177 journals that were added to the final ranking, although they did not fulfil the entrance criteria. These excluded journals were not subject to the expert-based evaluation.

Finally, we have analysed 2035 (92%) journals, including 679 in the H, 647 in the SS, and 709 in the ENM.

### Variables

We used nine measured (continuous) variables and one latent variable (i.e., construct for specifying models that allow us to estimate a number of points assigned to a journal in three fields of science).

*Internationalisation of authors*—percentage of authors from foreign countries (outside Poland).

*Internationalisation of reviewers*—percentage of reviewers from foreign countries (outside Poland).

*Internationalisation of publication language*—percentage of articles published in English, German, French, Spanish, Russian, and Italian.

*Internationalisation of editorial advisory board*—percentage of members in the editorial board from foreign countries (outside Poland).

*Age*—age of journal.

*PIF*—Predicted Impact Factor.

*Expert*-*based evaluation*—points obtained for the expert-based evaluation.

*Electronic version*—percentage of articles available in the electronic version. This indicator has been interpreted by the majority of journals as providing Internet access to articles published in the evaluated period (i.e., in the last two years). Nearly half of all Polish journals are open-access journals. This means that for many journals, an electronic version is identified with open access to articles.

*Points*—the final evaluation of the multidimensional evaluation in terms of the points.

*Journal internationalisation*—a latent variable (construct) is formed by the following measured variables: the *internationalisation of authors*, the *internationalisation of reviewers,* the *internationalisation of publication language*, and the *internationalisation of the editorial advisory board.* Such a construct was discussed in various publications. As an indicator of journal internationalisation, the following indicators were suggested: publication language (Buela-Casal et al. 2006; Rey-Rocha and Martin-Sempere 2004), author(s) affiliation (Buela-Casal et al. 2006; Gutiérrez and López-Nieva 2001; He and Liu 2009; Rey-Rocha and Martin-Sempere 2004; Uzun 2004; Wormell 1998; Yue and Wilson 2004), reviewer(s) affiliation (Buela-Casal et al. 2006; Pajić and Jevremov 2014), international cooperation (Glänzel and de Lange 2002; Zitt and Bassecoulard 1998), editorial board geographic location (Buela-Casal et al. 2006; Gutiérrez and López-Nieva 2001; He and Liu 2009; Rey-Rocha and Martin-Sempere 2004; Uzun 2004; Yue and Wilson 2004), international audience (Buela-Casal et al. 2006; Gutiérrez and López-Nieva 2001; He and Liu 2009; Rey-Rocha and Martin-Sempere 2004; Wormell 1998), and indexing in the bibliographic databases (Buela-Casal et al. 2006; Rey-Rocha and Martin-Sempere 2004). The limitations of the data collected during the multidimensional evaluation of Polish journals do not allow us to include an indicator related to indexing in the bibliographic databases. The gathered data (concerning a number of the databases in which a given Polish journal is indexed) do not differentiate the local and the international databases. Moreover, the dataset does not include the information about a geographical distribution of the readers or the international cooperation of authors. In these cited publications, authors also conducted their analyses on the basis of the limited number of indicators.

### Propositions

In this section, based on the literature review, we have suggested the following relationships between the variables:

1. *The journal internationalisation is related to the PIF in the ENM*: we suggest this association according to the relationship between the citation-based indicators and the internationalisation of journals. Yue and Wilson (2004) assume that a higher number of international authors and a higher number of editorial advisory board members increase a chance for the citations because such a relation makes a journal more attractive to the authors and readers. Zitt and Bassecoulard (1998; see also 2005) show that the relationship between the internationalisation indicators and the impact of journals from the engineering, natural, and medical sciences is average when it is measured by the citations. Bornmann et al. (2008) claim that the prestige of the journal may intensify the probability of citations. Dombrowski (1988) shows that there is a relationship between the citations and the publication language. According to Yue and Wilson (2004), this effect results from easier access to papers for readers from foreign countries. Gutiérrez and López-Nieva (2001) present a positive correlation between the journal internationalisation and the Impact Factor in the so-called hard sciences and a few of the social sciences (e.g. psychology). Conversely, there is a negative and low correlation in other social sciences (e.g., geography of political sciences).
2. *The PIF is related to the expert*-*based evaluation in the ENM*: we suggest this association according to the discussion concerning the relationship between two approaches: the citation-based indicators and the peer review. In various studies, the approaches are called the objective (citation-based indicators) and the subjective (expert-based evaluation). Kao (2009) states that if a journal publishes articles that are the object of discussion more often than articles from other journals and if, as a consequence, the journal in question has a higher Impact Factor, then this journal is acknowledged as a better quality journal. Yue and Wilson (2004) show that the journal quality may affect the journal citation impact. This study was based on Anderson and Goldstein’s work (1981, as cited in Yue and Wilson 2004), in which the authors presented the relationship between the journal reputation and the journal citations. Furthermore, Singleton (1976), Reale et al. (2007), and Vieira et al. (2014) demonstrated a positive correlation between the peer review and the citation-based indicators.
3. *The journal internationalisation is related to the expert*-*based evaluation in the H, the SS, and the ENM*: we suggest this association according to the discussion about the relationship between the degrees of internationalisation of scientific journals with the peer review. Yue and Wilson (2004) show that the internationalisation is a predictor of peer perception. Hicks and Wang (2010) show that at least two indicators (language and country) are appropriate to the journal evaluation. It allows us to assume that an expected relationship will occur between the *expert*-*based evaluation* and these two variables: the *internationalisation of authors* and the *internationalisation of publication language*.
4. *Age is related to the expert*-*based evaluation in the H and the SS*: we suggest this association according to the discussion about the relationship between journal age and journal reputation. Nazim Ali et al. (1996) suggest that the authors and readers should use journal age as a quality indicator of publishing continuity. Hicks and Wang (2010) show age as an indicator of journal perception in the scholarly community of the H and the SS.
5. *The electronic version affects the points in the H, the SS, and the ENM:* the *electronic version* is discussed as an indicator of the global access to a journal (Buela-Casal et al. 2006). Thus, we suggest that the *electronic version* is a predictor of the *points*. Moreover, this prediction is justified by the 2015 PJR regulations that prefer journals concerned with internationalisation and reputation.
6. *Journal internationalisation, PIF,* and *expert*-*based evaluation affect the points in the H, the SS, and the ENM*: we use the indicators (such as j*ournal internationalisation, PIF, and expert*-*based evaluation)* as the predictors of the *points*. This results from the 2015 PJR regulations and the current discussions about these indicators in the literature. In contrast to the H and the SS, we do not use *age* in the ENM because the impact of *age* on *points* is not significant.
7. *Journal internationalisation, expert*-*based evaluation, and age affect the points in the H and the SS*: we use the indicators (such as *journal internationalisation, expert*-*based evaluation,* and *age)* as predictors of the *points*. This results from the 2015 PJR regulations and the current discussions about these indicators in the literature. In contrast to the ENM, we do not use the PIF in the H and the SS. As various bibliometric analyses based on the Social Science Citation Index have shown, there is a relationship in the social sciences between the journal internationalisation and the citations, and there is also a relationship between the peer review and the citation, as in the ENM. In the case of Polish local journals, however, PIF plays no significant role in journal evaluation in the H and the SS. As Table 3 shows, the value of PIF equals 0 for 52% of journals, both in the H and the SS. Moreover, as presented in Table 2, journals from the H and the SS could obtain fewer points than journals from the ENM because of PIF. Thus, PIF affects the *points* (i.e., the final result of evaluation) in nonsignificant ways. Furthermore, the relationship between the PIF and the *internationalisation journal,* and the relationship between the PIF and the *expert*-*based evaluation,* are not significant. In the H and the SS, we use *age* as a significant variable that affects the *expert*-*based evaluation,* and we use *age* as a predictor of the *points*.

The propositions of associations are limited by the dataset character that is used in the present study and the ways in which the parameters of evaluation were measured. Table 4 presents the descriptive statistics of the above-defined measured variables and Table 5 presents the correlation matrix between these variables.

**Table 4:** Descriptive statistics of the measured variables according to three fields of science

Measured variables	Humanities			Social sciences			Engineering, natural and medical sciences		
	M	Me	SD	M	Me	SD	M	Me	SD
Internationalisation of authors	18.54	10	21.91	14.25	7	19.018	18.09	7	24.42
Internationalisation of reviewers	18.82	8	25.1	16.46	6	22.162	21.35	9	27.444
Internationalisation of publication language	26.5	9	35.169	26.22	11	33.688	46.44	25.5	44.818
Internationalisation of editorial advisory board	38.78	38.89	23.947	40.66	42.86	24.442	41.33	50	26.871
Age	19.35	11	20.65	15.87	10	16.343	26.34	18	23.815
PIF	.078	0	.225	.074	0	.241	1.158	.314	3.352
Expert-based evaluation	1.754	1	1.97	1.79	1	1.852	1.953	1	1.893
Electronic version	84.77	100	33.456	92.18	100	24.762	93.31	100	23.06
Points	7.87	7	3.19	8.08	8	3.284	8.14	8	3.438

**Table 5:** Correlation matrix between the measured variables according to the three fields of science

Measured variables	Field of science	1	2	3	4	5	6	7	8	9
1. Internationalisation of authors	H	–	.71**	.61**	.48**	−.13**	.2**	.13**	.01	.18**
	SS	–	.66**	.63**	.46**	−.13**	.29**	.12**	.08*	.22**
	ENM	–	.72**	.56**	.45**	−.07	.18**	.29**	.11**	.41**
2. Internationalisation of reviewers	H	.71**	–	.61**	.5**	−.13**	.18**	.14**	.08*	.22**
	SS	.66**	–	.6**	.48**	−.1*	.24**	.18**	.12**	.32**
	ENM	.72**	–	.57**	.5**	−.12**	.14**	.28**	.15**	.39**
3. Internationalisation of publication language	H	.61**	.61**	–	.39**	−.09*	.17**	.13**	.09*	.23**
	SS	.63**	.6**	–	.49**	−.1*	.3**	.23**	.16**	.36**
	ENM	.56**	.57**	–	.51**	−.02	.16**	.4**	.22**	.57**
4. Internationalisation of editorial advisory board	H	.48**	.5**	.39**	–	−.11**	.07	.16**	.15**	.29**
	SS	.46**	.48**	.49**	–	−.15**	.16**	.18**	.2**	.32**
	ENM	.45**	.5**	.51**	–	−.03	.08*	.31**	.16**	.47**
5. Age	H	−.13**	−.13**	−.09*	−.11**	–	.37**	.32**	−.13**	.36**
	SS	−.13**	−.1	−.1	−.15**	–	.26**	.39**	−.04	.38**
	ENM	−.07	−.12**	−.02	−.03	–	.14**	.21**	.01	.31**
6. PIF	H	.2**	.18**	.17**	.07	.37**	–	.31**	−.11**	.29**
	SS	.29**	.24**	.3**	.16**	.26**	–	.32**	.04	.37**
	ENM	.18**	.14**	.16**	.08*	.14**	–	.27**	.04	.29**
7. Expert-based evaluation	H	.13**	.14**	.13**	.16**	.32**	.31**	–	0	.83**
	SS	.12**	.18**	.23**	.18**	.39**	.32**	–	.07	.86**
	ENM	.29**	.28**	.4**	.31**	.21**	.27**	–	.13**	.87**
8. Electronic version	H	.01	.08*	.09*	.15**	−.13**	−.11**	0	–	.22**
	SS	.08*	.12**	.16**	.2**	−.04	.04	.07	–	.21**
	ENM	.11**	.15**	.22**	.16**	.01	.04	.13**	–	.27**
9. Points	H	.18**	.22**	.23**	.29**	.36**	.29**	.83**	.22**	–
	SS	.22**	.32**	.36**	.32**	.38**	.37**	.86**	.21**	–
	ENM	.41**	.39**	.57**	.47**	.31**	.29**	.87**	.27**	–

*H* Humanities, *SS* Social sciences, *ENM* Engineering, natural and medical sciences

* *p* < .05, ** *p* < .01

### Data analysis

In our analysis, we have used a regression-based structural equation modelling (SEM). SEM is a confirmatory approach to data analysis that allows for the specification and testing of theoretical (hypothetical) models that include various latent variables, multiple indicators, measurement errors, and complex structural relationships (Heck and Thomas 2015). In our research, structural models have been developed on the basis of the works in journal evaluation and the previous empirical findings. We have used the maximum likelihood as a method of estimation. The data were analysed with Mplus 7 (Muthén and Muthén 2015). Theoretical models are specified based on hypotheses (propositions). According to Hoyle (2012), the identification of these models is possible only when the number of identified parameters is lower than the number of variances and covariances in the matrix. This statistical limitation does not allow us to estimate all possible relations between the variables in our models.

The model fit was tested with the Root Mean Square Error Approximation (RMSEA), Standardised Root Mean Square Residual (SRMR), and the Comparative Fit Index (CFI). We have assumed the following recommended values of the model fit indicators: RMSEA < .05 (very good fit) or <.1 (reasonable fit) (Steiger 1989, as cited in Fan et al. 1999), SRMR < .08 (good fit) (Hu and Bentler 1999), and CFI > .95 (Lance et al. 2006). We decided not to include the *Χ* ^2^ goodness of fit test in the results. According to Barrett (2007), when there is a sample with many observations, and an interpretation is based on the *Χ* ^2^ test result, then the fitting model may be incorrectly rejected as’not-fitting‘. We have reported the values for all indicators, but we have interpreted only the values of RMSEA, SRMR, and CFI.

We analysed the data in three steps. First, a confirmatory factor analysis (CFA) was performed to determine whether the measured variables reliably reflect the hypothesised latent variable (i.e., the *journal internationalisation*). Second, we tested the paths in three full theoretical models designed separately for three fields of science: Model H, Model SS, and Model ENM. Finally, we removed one measured variable (i.e., the *expert*-*based evaluation*) from these models. In this way, the reduced (nested) models (i.e., Model H′, Model SS′, and Model ENM’) were constructed. We then compared each full model with its nested model to investigate which best explained a variation in the covariance matrix (e.g., we compared Model H with Model H′). The *Χ* ^2^ difference test was used to analyse whether the improvement in the model fits were significant.

$${\rm X}^{2}_{\text{difference}} = {\rm X}^{2}_{\text{full}} {-}{\rm X}^{2}_{\text{reduced}}$$

$$df_{\text{difference}} = df_{\text{full}} {-} \, df_{\text{reduced}}$$

R^2^ was used for testing what percentage of the variation in the values of the *points* (the dependent variable) could be explained by the full models (i.e. variables that affect the *points* in the models with the *expert*-*based evaluation*) and by the reduced models (i.e., variables that affect the *points* in the models without the *expert*-*based evaluation*).

## Results

### CFA

In the first step of the data analysis, we tested the reliability of the latent variable (construct): the *journal internationalisation*.

We tested whether the four variables (the *internationalisation of authors*, the *internationalisation of reviewers,* the *internationalisation of publication language*, and the *internationalisation of the editorial advisory board*) constitute a defining part of the construct (i.e., we tested if there are correlations between the variables and the construct). According to Tabachnick and Fidell (2007), we have assumed that a variable is relevant for the particular construct when a minimal value of the standardised factor loading is.32. Table 6 presents the values of the standardised factor loadings and the standard errors. All four variables related to the internationalisation are significantly related to the construct (i.e., the *journal internationalisation*). This result confirms that including these variables in the next step of our analysis is valid.

**Table 6:** Standardised factor loadings for the factor confirmatory model of the *Journal Internationalisation* according to the fields of science

Field of science	Indicator name	Standardised factor loading	SE
Humanities	Internationalisation of authors	.842***	.017
	Internationalisation of reviewers	.85***	.017
	Internationalisation of publication language	.719***	.022
	Internationalisation of editorial advisory board	.572***	.029
Social sciences	Internationalisation of authors	.781***	.027
	Internationalisation of reviewers	.761***	.029
	Internationalisation of publication language	.801***	.025
	Internationalisation of editorial advisory board	.614***	.029
Engineering, natural and medical sciences	Internationalisation of authors	.712***	.029
	Internationalisation of reviewers	.747***	.028
	Internationalisation of publication language	.773***	.026
	Internationalisation of editorial advisory board	.658***	.028

*** *p* < .001

The construct reliability is high: Cronbach’s alfa coefficient is *α* = .813 for the H, *α* = .813 for the SS, and *α* = .803 for the ENM. We have conducted CFA to test construct reliability. The analysis has confirmed that the construct structures are identified in three fields of science: H: *χ* ^2^ = 2.196, *df* = 2, *p* > .05, CFI = 1, RMSEA = .012; SS: *χ* ^2^ = 2.018, *df* = 1, *p* > .05, CFI = .999, RMSEA = .04; ENM: *χ* ^2^ = 2.176, *df* = 1, *p* > .05, CFI = .999, and RMSEA = .041. The high CFI indicates that the construct structure is a valid one.

### Evaluation of the models

In the second step, we have investigated how the theoretical (hypothetical) models designed for each field of science fit the data. We have tested the full model and the reduced model (without the *expert*-*based evaluation*) for each field of science. Next, we have compared the full model with the reduced one.

#### Model H

**Fig. 1:**
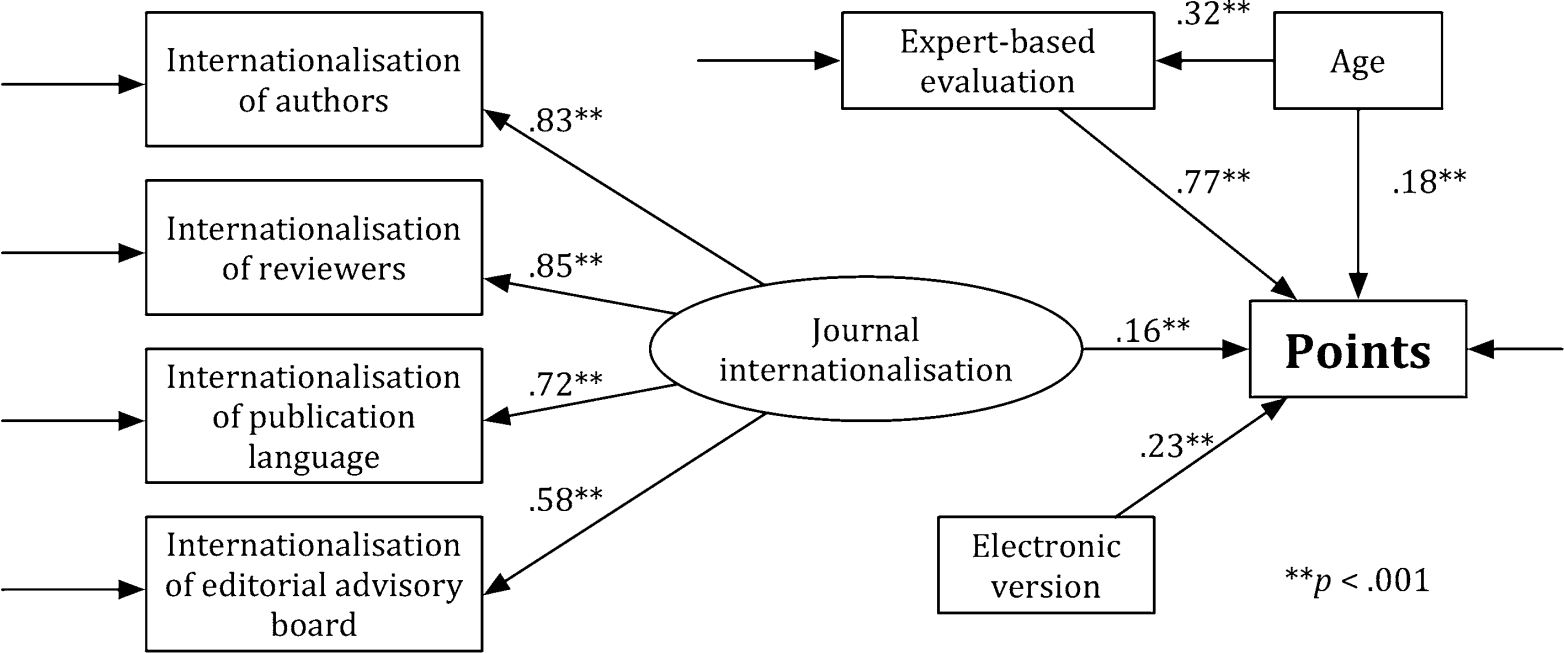
Model H

Figure 1 presents Model H that is specified for the humanities (H). Although the value of the general test for the fit was significant, *χ* ^2^ = 109.387, *df* = 18, *p* < .05, the values of other indicators, which were used for estimating the goodness of the model fit, show that the fit of Model H to the data is acceptable and that RMSEA = .086, CFI = .96, and SRMR = .075. The analysis of the significance of path coefficients has revealed that all associations in the theoretical model were significant (*p* < .001). The *journal internationalisation* (*b* = .156, SE = .021, *p* < .001), the *expert*-*based evaluation* (*b* = .771, SE = .016, *p* < .001), the *age* (*b* = .178, SE = .021, *p* < .001), and the *electronic version* (*b* = .231, SE = .02, *p* < .001) were predictors of the *points*. The *age* was a predictor of the *expert*-*based evaluation* (*b* = .317, SE = .035, *p* < .001). Model H explains 76% of the point variability (*R* ^2^ = .766).

**Fig. 2:**
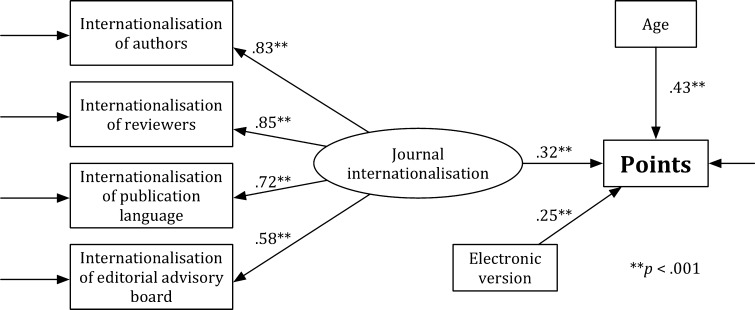
Model H′

Figure 2 presents model H′. As in Model H, a value of the general test for the fit was significant, *χ* ^2^ = 65.241, *df* = 13, *p* < .05. The other indicators show that the fit of Model H′ to the data is acceptable: RMSEA = .077, CFI = .962, and SRMR = .057. Analysis of the significance of path coefficients revealed that all associations in the theoretical model were significant (*p* < .001). The *journal internationalisation* (*b* = .316, SE = .033, *p* < .001), the *age* (*b* = .433, SE = .029, *p* < .001), and the *electronic version* (*b* = .248, SE = .032, *p* < .001) were predictors of the *points*. Model H′ explains 32% of the point variability (*R* ^2^ = .321).

The *χ* ^2^ difference test for the comparison of Model H with Model H′ is significant, *χ*_difference_^2^ = 44.146, *df*_difference_ = 5, and critical value for a 5 *df* is 11.07 (*p* < .05). Model H (i.e., the full model) is more acceptable than the reduced model (i.e., Model H′). Moreover, Model H better explains the points variability (76%) than Model H′ (32%). Comparison of the models with and without the expert-based evaluation shows that the expert-based evaluation significantly influenced the results of the multidimensional evaluation in the H.

#### Model SS

**Fig. 3:**
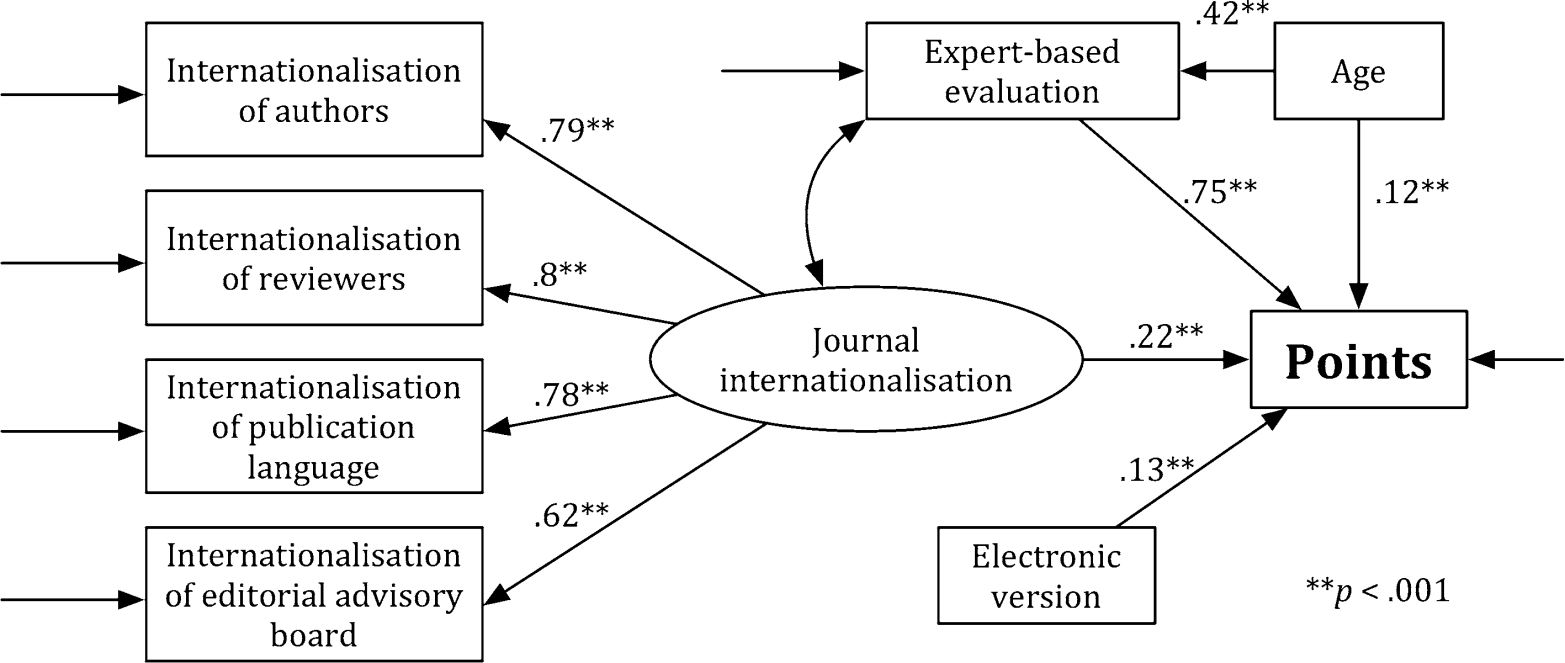
Model SS

Figure 3 presents Model SS, which is specified for the social sciences (SS). As in Model H, a value of the general test for the fit was significant, *χ* ^2^ = 103.681, *df* = 17, *p* < .05; the values of other indicators, which were used for estimating the goodness of the model fit, show that the fit of Model SS to the data is acceptable: RMSEA = .089, CFI = .962, and SRMR = .068. The analysis of the significance of path coefficients has revealed that all associations in the theoretical model were significant (*p* < .001). The *journal internationalisation* (*b* = .216, SE = .021, *p* < .001), the *expert*-*based evaluation* (*b* = .753, SE = .018, *p* < .001), the *age* (*b* = .121, SE = .02, *p* < .001), and the *electronic version* (*b* = .129, SE = .018, *p* < .001) were predictors of the *points*. The *age* was a predictor of the *expert*-*based evaluation* (*b* = .417, SE = .031, *p* < .001). The *journal internationalisation* was associated with the *expert*-*based evaluation* (*b* = .317, SE = .039, *p* < .001). Model SS explains 81% of the points variability (*R* ^2^ = .81).

**Fig. 4:**
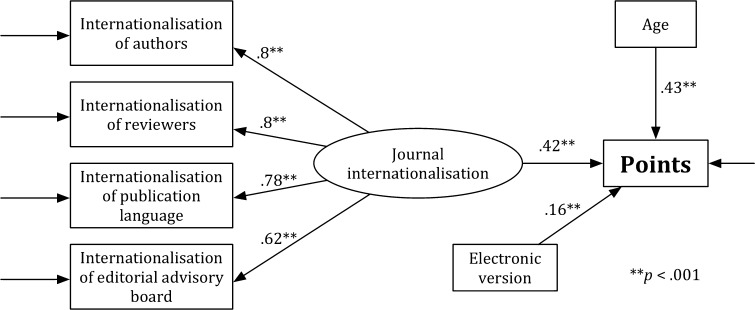
Model SS′

Figure 4 presents Model SS’. As in Model SS, a value of the general test for the fit was significant, *χ* ^2^ = 93.812, *df* = 13, *p* < .05. The values of indicators RMSEA = .098, CFI = .94, and SRMR = .071 show that the fit of Model SS’ to the data is acceptable, but the value of indicator CFI = .94 is slightly below the acceptable limit. Thus, the model should not be accepted. Analysis of the significance of path coefficients revealed that all associations in the theoretical model were significant (*p* < .001). The *journal internationalisation* (*b* = .425, SE = .032, *p* < .001), the *age* (*b* = .435, SE = .029, *p* < .001), and the *electronic version* (*b* = .162, SE = .032, *p* < .001) were predictors of the *points*. Model SS’ explains 39% of the points variability (*R* ^2^ = .39).

The *χ* ^2^ Chi square difference test for the comparison of Model S with Model SS’ is significant, *χ*_difference_^2^ = 9.869, *df*_difference_ = 4, and the critical value for a 4 *df* is 9.488 (*p* < .05). Model SS (i.e., the full model) is more acceptable than the reduced model (i.e., Model SS’). Moreover, Model SS better explains the points variability (81%) than Model SS’ (39%). Comparison of the models with and without the expert-based evaluation shows that the expert-based evaluation significantly influenced the results of the multidimensional evaluation in the SS.

#### Model ENM

**Fig. 5:**
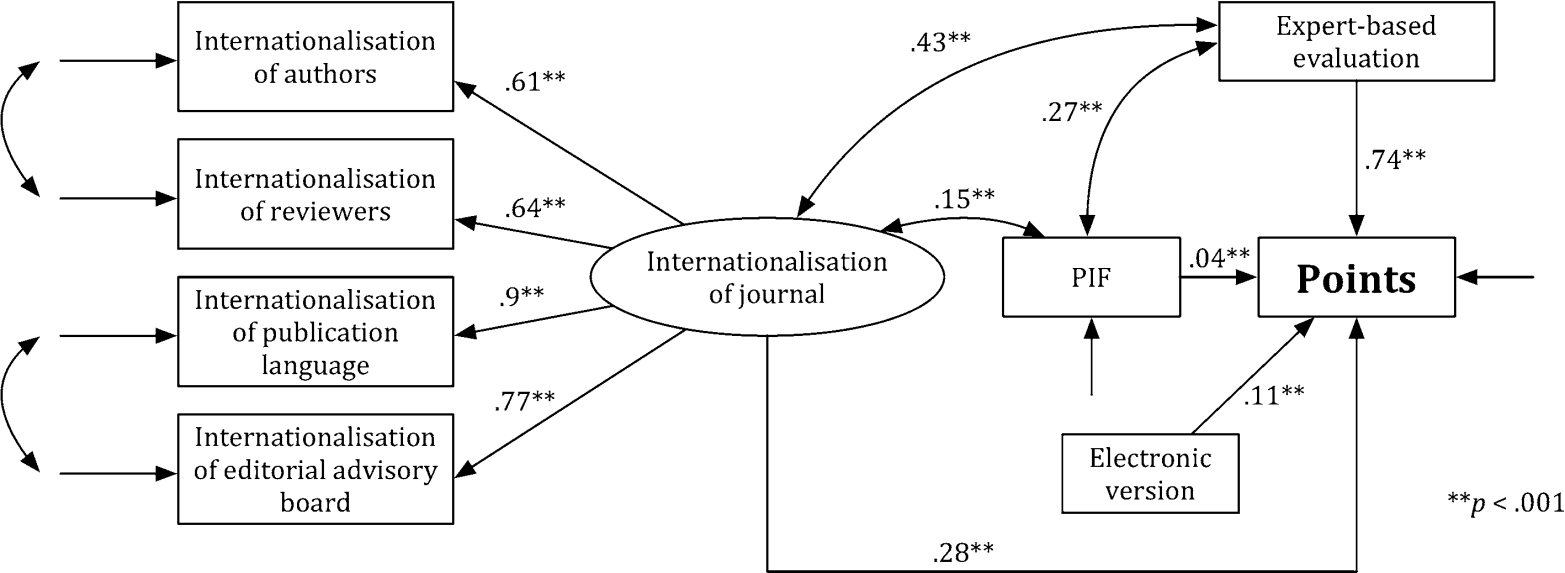
Model ENM

Figure 5 presents the Model ENM that is specified for the engineering, natural, and medical sciences (ENM). As in Models H and SS, a value of the general test for the fit was significant, *χ* ^2^ = 59.293, *df* = 15, *p* < .05, and the values of other indicators, which were used for estimating the goodness of the model fit, show that the fit of Model ENM to the data is acceptable: RMSEA = .065, CFI = .983, and SRMR = .061. The analysis of the significance of path coefficients has revealed that all associations in the theoretical model were significant (*p* < .001). The *journal internationalisation* (*b* = .283, SE = .021, *p* < .001), the *expert*-*based evaluation* (*b* = .736, SE = .017, *p* < .001), the PIF (*b* = .045, SE = .016, *p* < .01), and the *electronic version* (*b* = .111, SE = .016, *p* < .001) were predictors of the *points*. The *journal internationalisation* was associated with the PIF (*b* = .154, SE = .039, *p* < .001) and the *expert*-*based evaluation* (*b* = .0434, SE = .034, *p* < .001). The PIF was associated with the *expert*-*based evaluation* (*b* = .273, SE = .035, *p* < .001). Model ENM explains 84% of the points variability (*R* ^2^ = .839).

**Fig. 6:**
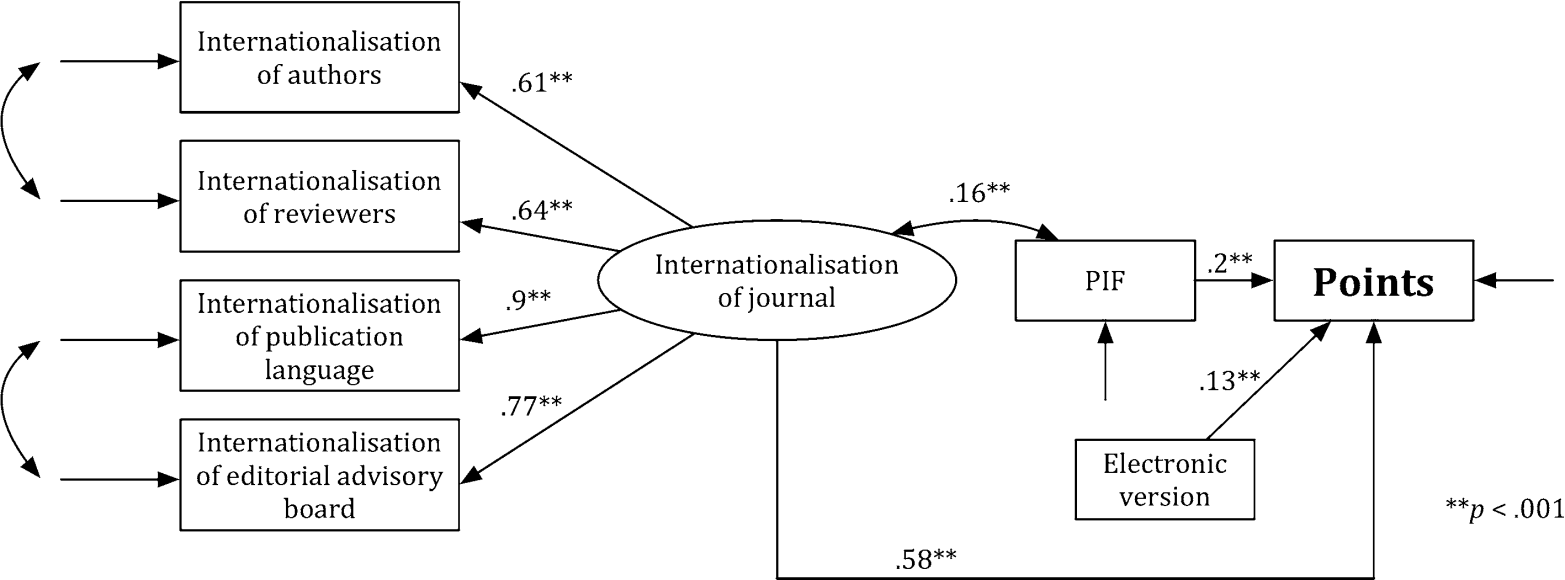
Model ENM′

Figure 6 presents the Model ENM′. A value of the general test for the fit was significant, *χ* ^2^ = 53.098, *df* = 11, *p* < .05. The other indicators show that the fit of Model ENM’ to the data is acceptable, RMSEA = .073, CFI = .973, and SRMR = .063. Analysis of the significance of path coefficients revealed that all associations in the theoretical model were significant (*p* < .001). The *journal internationalisation* (*b* = .577, SE = .03, *p* < .001), the PIF (*b* = .198, SE = .03, *p* < .001), and the *electronic version* (*b* = .134, SE = .03, *p* < .001) were predictors of the *points*. The *journal internationalisation* was associated with the PIF (*b* = .158, SE = .039, *p* < .001). The model ENM′ explains 43% of the points variability (*R* ^2^ = .427).

The χ^2^ Chi square difference test for the comparison of Model ENM with Model ENM’ is not significant, *χ*_difference_^2^ = 6.195, *df*_difference_ = 4, critical value for a 4 *df* is 9.488 (*p* < .05). Thus, Model ENM does not significantly differ from Model ENM’, and it is fit to the data in the same way as Model ENM′. However, Model ENM better explains the points variability (84%) than Model ENM’ (43%). Comparison of the model with and without the *expert*-*based evaluation* shows that—contrary to the results in the H and the SS—the *expert*-*based evaluation* did not significantly influence the results of the multidimensional evaluation in the ENM. However, we have included the PIF only in the ENM and ENM’ models. This decision follows the assumption that we construct models in line with the previous empirical works. It is noteworthy that the correlation coefficients between the PIF and the *expert*-*based evaluation* are also very similar in all fields. Hence, including the PIF in the ENM model might provide an alternative explanation as to why there is no effect for the *expert*-*based evaluation* in ENM. However, such an explanation cannot be based on the models built according the SEM’s methodological and theoretical assumptions.

In our analysis, we have designed three pairs of theoretical models for three fields of science: the H, the SS, and the ENM. Each pair consists of the full model (e.g., Model H for the H) and the reduced model; that is, the model without the *expert*-*based evaluation* (e.g., Model H′ for the H). We have confirmed that our propositions are significant. At the same time, we have presented no results of any alternative models that could be built on the basis of relations identified by the descriptive statistics. According to the SEM, we have worked on the basis of propositions constructed in line with the previous empirical works.

Furthermore, the model comparison has shown that the full model significantly differs from the nested one in the H and the SS. Model H and Model SS are better fit to the data than Model H′ and Model SS′.

Moreover, the full model does not significantly differ from the nested one in the ENM. Therefore, if we decide to eliminate the expert-based dimension in the multidimensional evaluation of national journals in the ENM, both the full and nested models are acceptable. It means that Model ENM and Model ENM′ are well fit to the data.

However, the analysis of R^2^ shows that the full models explain the *points* variability in a better way in all three fields of science: H, SS, and ENM. It shows that the expert-based dimension of the multidimensional evaluation of national journals is significant, regardless of the field of science.

## Discussion and conclusion

In conclusion, our findings show that the multidimensional ranking of local journals should not be constructed only on the basis of the bibliometric evaluation. In the Polish case, reducing the 2015 PJR dimensions to the bibliometric dimension could keep 52% of journals from the H and the SS from being included in the ranking. According to our analysis, this dimension is significant only to the ENM in the multidimensional evaluation of national journals.

As presented, the expert-based dimension plays an important role in multidimensional evaluation. Theoretical models that include this dimension explain the majority of the variability of results evaluation in all fields of science.

However, when we look at the interrelationships between the indicators in the analysed models, we can see that including the expert-based dimension has increased the model fitting in the H and the SS models, but this effect is not observed with respect to the ENM.

The expert-based evaluation has been introduced in the current journal evaluation system, as requested by the majority of Polish scholars. However, the procedure of the evaluation itself was not properly designed and should be improved in many aspects. The experts might be affected by the previous evaluation of journals because they could check if a journal was previously assessed and how many points were assigned. The experts did not know how journals were evaluated in formal and bibliometric evaluation in the current cycle of assessment. Moreover, the experts, who received too many journals for assessment may have experienced the halo effect. According to Serenko and Dohan (2011), the extremely high or low quality of a preceding journal may affect quality perceptions of the subsequent journals. There are many other limitations of the expert-based evaluation (e.g. experts’ research interests or the time and costs of implementation). Thus, the procedures should be continuously improved so these valuable resources of academic community will not be wasted.

Development of the PJRs is possible but should be implemented gradually to make it possible to assess and redesign a new version of this journal-evaluation system. According to our findings, we suggest four main steps that could improve the current solution and may be relevant for the journal evaluation systems in other non-English countries.

The first two suggestions are relevant for the journal evaluation from all fields of science: (1) the formal evaluation should be reduced only to such parameters that could be verified by the STESJ members: the current solution is based mostly on the trust in the data provided by the journal editors and is perceived by many scholars and stakeholders as insufficient; (2) the expert-based evaluation should be conducted on the basis of some common guidelines for the experts and, moreover, the committees should provide the internal regulations for their experts that might be adjusted for various fields of science.

The next two suggestion are especially important for the social sciences and the humanities: (3) Non-Polish journals that are indexed neither in the WoS nor the ERIH should also be indexed in the *B list*: the current solution does not allow for keeping the balance between the humanities and other fields of science; (4) in the next cycles of PJRs, improved bibliometric indicators should be used that fit to publication languages, different disciplines, and publication patterns: one of the possible solutions is to re-define the announced *Polish Impact Factor* and to collect the bibliographic data of books that are not only Polish in the POL-index. Currently, only references from articles have been gathered.

In our analysis, we have focused on showing how adding the expert-based dimension affected the results in the three science groups. Nonetheless, the data and the relations between the variables presented in the present paper might be an important component in the discussion concerning the multidimensional journal evaluation in general. For instance, in Table 5, one can see that there is no correlation or only a weak correlation between the *electronic version* and the variables constituting journal internationalisation. However, international databases (e.g., Web of Science Core Collection and SCOPUS), which index journals with a high level of internationalisation, expect the electronic versions. This may be evidence that using indicators specified for international journals and relying on an electronic version in the evaluation of local journals may not be well grounded.

In some areas of science policy in Poland, the collection of points and use of PJRs in the academic-promotion procedures have become targets themselves. Analysis of such unintended uses could provide some new arguments regarding how to change the research-evaluation system in Poland. Thus, the aim of the PJRs’ improvement must be not only a good quality evaluation but for scientometricians and policy makers to take into account that each change in this system affects some new, unintended uses that should be reduced.
